# Editorial: Comprehensive treatment strategy for improving surgical resection rate of retroperitoneal sarcomas

**DOI:** 10.3389/fonc.2025.1687933

**Published:** 2025-09-11

**Authors:** Hanxing Tong

**Affiliations:** Department of General Surgery, Zhongshan Hospital, Fudan University, Shanghai, China

**Keywords:** retroperitoneal sarcomas, diagnosis, surgical technique, perioperative care, systemic therapy

Retroperitoneal sarcomas (RPS) present a formidable challenge in surgical oncology. Their rarity, anatomical complexity, frequent involvement of critical structures, and pronounced histological heterogeneity contribute to high rates of local recurrence and significant morbidity, despite aggressive treatment. This Research Topic brings together a collection of insightful studies that collectively advance our understanding of RPS management, highlighting significant progress and persistent challenges across the spectrum of diagnosis, surgical technique, perioperative care, staging, and systemic therapy. The contributions underscore the critical importance of a multidisciplinary, histology-tailored approach to this complex disease.

The cornerstone of curative intent for localized RPS remains complete surgical resection with negative margins (R0). Achieving this goal often necessitates complex, extended procedures. The case series by Al-Makassed Hospital powerfully illustrates this reality, demonstrating successful R0 resection in three patients with locally advanced sarcomas invading major vessels, kidneys, and other viscera (Bael et al.). These successes were unequivocally attributed to meticulous preoperative planning and the indispensable role of a dedicated, highly specialized multidisciplinary surgical team encompassing vascular, urologic, and hepatobiliary expertise. This reinforces the paradigm that complex RPS surgery demands a collaborative effort far beyond the scope of a single surgeon. Complementing this, the comparative study on Total Retroperitoneal Lipectomy (TRL) provides compelling evidence that extending the resection beyond the tumor mass itself, specifically in primary retroperitoneal liposarcoma (RPLS) involving the removal of ipsilateral retroperitoneal fat, improves recurrence-free survival (RFS) and overall survival (OS) compared to traditional complete resection (CR), particularly in dedifferentiated subtypes, without significantly increasing severe morbidity (Gao et al.). This suggests a potential shift towards more extensive compartmental resections for specific histologies.

Technological advancements continue to refine surgical approaches. The large single-center experience with Da Vinci robot-assisted retroperitoneal tumor resection (RRTR) demonstrates the safety and efficacy of this minimally invasive technique in selected patients (Hao et al.). Their analysis identified a maximum tumor diameter ≤64 mm and benign pathology as key predictors of successful robotic resection, offering valuable practical guidance for patient selection and setting the stage for further refinement of robotic applications in RPS. While successes in R0 resections and robotic approaches are well highlighted, a brief acknowledgment of limitations, as most existing reports are single-center, retrospective, or small sample sizes. Therefore, future exploration through large-scale cohort studies or even prospective studies is warranted.

The complexity of RPS surgery inherently carries significant perioperative risks. The study on anesthetic management in patients requiring massive blood transfusion (MBT) during RPS resection provides crucial insights into mitigating these risks (Wang et al.). It highlights the profound blood loss encountered (median 7000ml) and the extensive transfusion requirements, while identifying elevated lactate levels at surgery’s end as the sole significant risk factor for perioperative mortality. This underscores the critical importance of meticulous intraoperative hemodynamic monitoring, aggressive resuscitation strategies, and vigilant postoperative care in this high-risk cohort.

Accurate diagnosis and prognostication are of utmost importance. The analysis of retroperitoneal unicentric Castleman disease (UCD) resected as sarcomas serves as a crucial reminder of diagnostic pitfalls in the retroperitoneum (Gao et al.). Although UCD is exceptionally rare, its potential to mimic sarcoma necessitates awareness, as complete surgical resection offers excellent long-term survival. Conversely, the comprehensive evaluation of the AJCC staging system and proposal of a novel stage grouping system (nTNM) for RPLS addresses the limitations of current staging (Fan et al.). This large study, which combines institutional and SEER data, convincingly demonstrates that the revised T-stage and nTNM system provide superior risk stratification compared to the 7th and 8th AJCC editions. Furthermore, the developed nomogram offers a valuable tool for individualized prognostic assessment and treatment planning.

The role of adjuvant and neoadjuvant therapies in RPS remains an area of active investigation and debate. The population-based propensity score-matched study on intraoperative radiotherapy (IORT)delivers a sobering message, finding no significant improvement in overall survival for patients with RPLS receiving IORT compared to those who did not (Zhou et al.). This suggests IORT should not currently be considered a standard therapy outside of clinical trials, emphasizing the need for more effective local control strategies. In the realm of systemic therapy for advanced disease, the retrospective study on Anlotinib combined with Envafolimab shows promising activity in unresectable or metastatic liposarcoma, achieving a disease control rate of 73.3% and a median progression-free survival of 14.2 months, with manageable toxicity (Liu et al.). This adds to the growing armamentarium of targeted and immunotherapeutic options being explored for specific sarcoma subtypes.

Looking towards the future, understanding the molecular drivers of RPS is essential for developing novel therapeutics. The review “*Targeting Liposarcoma: Unveiling Molecular Pathways and Therapeutic Opportunities*” synthesizes the critical molecular heterogeneity across liposarcoma subtypes, detailing distinct genetic alterations and signaling pathways (Liu et al.). This knowledge is foundational for the rational design and application of histology-specific targeted therapies, moving beyond a one-size-fits-all approach (Garcia-Ortega). Similarly, the development and validation of the Comprehensive Hematological Scoring System (CHSS) represents a step forward in prognostication (Qiu et al.). By integrating multiple pre-treatment hematological markers, CHSS outperforms established ratios like NLR and PLR in predicting overall survival for soft tissue sarcoma patients, offering a potentially more robust tool for risk assessment.

In conclusion, the studies presented in this Research Topic paint a picture of a field in evolution. Significant strides are being made in refining surgical techniques, from the imperative of multidisciplinary radical resection to the exploration of minimally invasive robotics and extended compartmental procedures like TRL, guided increasingly by histological subtype. Perioperative care is being better defined, particularly for high-risk scenarios like massive transfusion. Diagnostic accuracy and prognostication are improving through awareness of mimics like UCD and the development of superior staging systems (nTNM) and prognostic scores (CHSS). While challenges remain, particularly in defining the optimal role for radiotherapy and overcoming the limitations of current systemic therapies, promising avenues are emerging, exemplified by the activity of anlotinib plus envafolimab and the growing understanding of subtype-specific molecular vulnerabilities. The overarching theme resonating throughout these contributions is the necessity for personalized, histology-driven management strategies delivered within a framework of close multidisciplinary collaboration. Continued research focusing on the molecular underpinnings of RPS subtypes and innovative therapeutic combinations holds the key to further improving outcomes for patients facing this challenging group of malignancies.

